# Addition of lenalidomide to melphalan in the treatment of newly diagnosed multiple myeloma: the National Cancer Institute of Canada Clinical Trials Group MY.11 trial

**DOI:** 10.3747/co.2007.107

**Published:** 2007-04

**Authors:** D.J. White, N. Paul, D.A. Macdonald, R.M. Meyer, L.E. Shepherd

**Affiliations:** * Division of Hematology, Queen Elizabeth ii Health Sciences Centre and Dalhousie University, Halifax, Nova Scotia; † The National Cancer Institute of Canada, Clinical Trials Group, Queen’s University, Kingston, Ontario

**Keywords:** Multiple myeloma, newly diagnosed, melphalan, thalidomide, lenalidomide

## Abstract

Oral melphalan and prednisone remain an effective and tolerable treatment for patients with multiple myeloma. For approximately 40 years, this combination has been the standard of care for patients not proceeding to stem cell transplant. Within the last 10 years, new agents have been found to be efficacious in the relapsed/refractory setting. Within the last year, two trials of added thalidomide in the newly diagnosed setting have demonstrated outcomes superior to those achieved with melphalan and prednisone alone. This improved outcome comes at the cost of increased toxicity.

The National Cancer Institute of Canada Clinical Trials Group (ncic ctg) has recently developed a randomized phase ii trial (MY.11) that uses a combination of lenalidomide with melphalan for patients with newly diagnosed multiple myeloma. Lenalidomide is a thalidomide analogue and, like thalidomide, is thought to work through immunomodulatory effects. It was shown to have activity in patients with relapsed or refractory disease and, in combination with dexamethasone, is superior to dexamethasone alone. Lenalidomide holds promise as a more effective and potentially less toxic derivative of thalidomide. Experience with lenalidomide in combination with chemotherapy is very limited, and the purpose of MY.11 is to establish tolerability and to gain knowledge about efficacy. The information gained from MY.11 is expected to help inform dosing levels and schedules for a large phase iii trial being developed by the Eastern Cooperative Oncology Group that will include participation by the ncic ctg.

## 1. INTRODUCTION

Multiple myeloma remains an incurable malignancy that causes significant morbidity in the people affected. Although myeloma accounts for only 1.0% of all malignancies, it accounts for approximately 13% of hematologic neoplasms, with an average annual age-adjusted incidence rate of 4.7 per 100,000 male population and 3.2 per 100,000 female population 1.

Before the introduction of effective chemotherapy with melphalan and prednisone, median overall survival in myeloma was approximately 17 months 2. The combination of melphalan and prednisone, introduced almost 40 years ago, provided an effective and well-tolerated regimen that was, until recently, considered the standard of care for myeloma patients not planned for transplantation. Initial response rates for the regimen approach 70%, but fewer than 5% of patients achieve a complete remission. Median overall survival is just 3.5 years, and 35 years of testing have failed to produce a superior chemotherapy regimen. But in 2006, two randomized controlled trials were reported that demonstrated superior outcomes in patients treated with melphalan, prednisone, and thalidomide^3^,^4^.

Palumbo and colleagues published the results of a myeloma trial completed by the Italian Cooperative Group. It demonstrated that, in older patients with myeloma (median age: 72 years), the 2-year event-free survival was superior in patients who received 100 mg thalidomide daily in addition to standard doses of melphalan and prednisone [54% vs. 27%; hazard ratio (hr): 0.51; 95% confidence interval (ci): 0.35 to 0.75; *p* = 0.0006]. Patients allocated to receive thalidomide also received that drug as maintenance therapy. The trial failed to detect a difference in overall survival (hr: 0.68; 95% ci: 0.38 to 1.22; *p* = 0.19), but the trial design did not have statistical power sufficient to address that outcome measure.

In the second trial, reported by Facon *et al.*, the French myeloma group completed a three-arm trial comparing melphalan and prednisone, intermediate-dose melphalan with stem cell support, and standard doses of melphalan and prednisone plus thalidomide at best tolerated dose to a maximum of 400 mg daily. Compared with melphalan and prednisone, the same agents with thalidomide added improved both median progression-free survival (29.5 months vs. 17.5 months, *p* < 0.001) and overall survival (not reached vs. 30.3 months, *p* = 0.0008).

The results from these two trials have made the combination of melphalan, prednisone, and thalidomide the standard of care for previously untreated patients with myeloma who are not candidates for autologous transplantation. Access to thalidomide in Canada and cost of the drug in Canada and in other countries remain barriers to common first-line use of this new combination.

In two randomized trials, the use of high-dose melphalan supported by blood stem cell transplantation was demonstrated to confer a survival advantage in younger patients^5^,^6^. Consistent results from other randomized trials comparing transplantation with standard-dose chemotherapy have not been observed: two recently published trials failed to detect differences in important outcomes^7^,^8^. Nevertheless, transplantation remains a standard option that is limited to younger patients who are able to tolerate dose-intensive therapy. Standard-dose therapy with melphalan, prednisone—and now thalidomide—is generally considered for patients of more advanced age or with comorbid illnesses.

Immunomodulatory drugs are a recent discovery in the treatment of myeloma. They have shown remarkable antimyeloma activity, particularly in the setting of relapsed or refractory disease. Thalidomide, the prototype of this group, was originally proposed to have an antitumour effect through inhibition of angiogenesis; more recent data suggest that the antitumour effect is mediated through several diverse mechanisms^9^.

Side effects with thalidomide include mild to moderate constipation, neuropathy, or somnolence in at least one third of patients^10^. In addition, thalidomide has been associated with risks of venous thromboembolism. Thus, testing of thalidomide analogues that have at least similar, if not superior, efficacy and a safer toxicity profile is desirable.

## 2. DISCUSSION

### 2.1 Lenalidomide in the Treatment of Myeloma

Lenalidomide is a derivative of thalidomide that has demonstrated anti-myeloma activity in phase i and ii studies evaluating patients with relapsed or refractory disease. Lenalidomide is not associated with significant constipation, neuropathy, or somnolence^11^–^13^. In addition, in a randomized trial evaluating patients with relapsed myeloma, a comparison of dexamethasone and lenalidomide with dexamethasone and placebo showed that patients in the lenalidomide-containing arm had superior outcomes even if they had received prior thalidomide^14^. These data suggest that, at a minimum, lenalidomide has some degree of non-cross-reactivity with thalidomide or potential as a more potent anti-myeloma agent. The drug therefore holds promise as an orally available, more effective, and less toxic derivative of thalidomide for the treatment of multiple myeloma.

### 2.2 The National Cancer Institute of Canada Clinical Trials Group MY.11 Trial

The experience evaluating lenalidomide in combination with melphalan has been limited, although both drugs have now been shown to be effective therapy in patients with myeloma. To evaluate the combination of these agents and to investigate the possibility of improved response rates and progression-free survival for previously untreated patients, the National Cancer Institute of Canada Clinical Trials Group (ncic ctg) has developed a phase ii trial, MY.11, that uses a combination of lenalidomide and melphalan in newly diagnosed patients. The tolerable dose of lenalidomide in combination with melphalan is not known. Because the expected toxicity of lenalidomide is myelosuppression, both short-term (cycles 1 and 2) and, just as importantly, longer-term (cycles 3–6 and possibly beyond) cumulative myelosuppression must be assessed to optimize the tolerable dose of lenalidomide and to maximize the potential for success of combination therapy in phase iii testing.

Other groups will be conducting traditional phase i trials with short-term toxicity endpoints, but ncic ctg believes that longer-term ability to deliver melphalan is also likely to be critical in the phase iii setting, and that the best way to establish tolerability over multiple cycles, while gaining valuable information about the efficacy of the combination, is to conduct a trial using a randomized phase ii design. The induction phase in the MY.11 study therefore uses a randomized phase ii design to investigate the effect of using lenalidomide in combination with melphalan as initial therapy in patients with multiple myeloma for whom high-dose melphalan and peripheral blood stem cell transplantation is not considered appropriate by virtue of age, comorbid illness, or patient choice. As an initial step, each arm of the proposed randomized phase ii trial will be tested separately in a consecutive manner to demonstrate safety before further testing is undertaken.

The concept of combining melphalan and lenalidomide is an attractive one. The ncic ctg are aware of two other groups who are also developing the concept.

The first is the myeloma team based at the Mayo Clinic, who are conducting a standard phase i study testing the addition of lenalidomide to the combination of melphalan and prednisone. Preliminary reports suggest that 5 mg/m^2^ melphalan and 60 mg/m^2^ prednisone daily for 4 days plus 10 mg lenalidomide on days 1–21 in a 28-day cycle will subsequently be tested in a phase ii trial^15^.

The second group of investigators, from Italy, is led by Palumbo. This group is conducting a phase i study testing the addition of lenalidomide to the combination of melphalan and prednisone. The most recent update of this trial^16^ reported the outcomes of 50 patients enrolled into four dose levels. The first two dose levels combine lenalidomide 5 mg daily for 21 days every 4–6 weeks with melphalan 0.18 mg/kg or 0.25 mg/kg given daily for 4 days. No dose-limiting toxicities have been observed.

In a third dose level that is testing lenalidomide 10 mg and melphalan 0.18 mg/kg, one dose-limiting toxicity (neutropenia) has been observed in the first 21 patients. Granulocyte colony stimulating factor (g-csf) was frequently required, and even with g-csf support, dose delays and attenuations were frequent. The overall response rate observed has been 100%, and 24% of patients achieved a complete remission.

A fourth dose level of melphalan 0.25 mg/kg and lenalidomide 10 mg is also being tested. At those doses, g-csf use and dose attenuations and delays have been more common. The overall response rate is 100%, and the complete remission rate is 10%.

Final determination of the data for the use of these agents remains to be completed, but the preferred doses are likely to be melphalan 0.18 mg/kg and lenalidomide 10 mg. That dose of melphalan translates to 7 mg/m^2^, pending the body habitus of the patient. Based on the preliminary data, use of these doses would anticipate regular use of g-csf to reduce the risks associated with profound neutropenia and to maintain the treatment schedule.

The initial intent of ncic ctg was to compare the standard dose of melphalan (9 mg/m^2^ daily for 4 days every 28 days) combined with lenalidomide at either 10 mg or 20 mg daily for 21 days every 28 days, with each arm to be tested in separate and consecutive safety run-ins in 6 patients before the randomized portion of the trial commenced. The MY.11 protocol was activated in December 2005, and by April 2006, 4 patients had been enrolled and had received their first cycles of therapy. Based on dose-limiting toxicity seen in the first 4 patients evaluated at the 10-mg lenalidomide dose, and taking into account the emerging data described above, accrual to the initial treatment protocol was suspended, and the drug doses redefined. The revisions now include reduced doses of melphalan and of lenalidomide, and a new, more restrictive, dose attenuation schedule that begins with dose attenuations on day 8 of each treatment cycle if features of myelosuppression are noted at that time. In addition, the eligibility criteria for the trial have been modified, and now include a need for normal platelet count upon study entry. The additional data from the Italian group indicate a high likelihood of initial tolerability of the new doses to be tested.

The revised MY.11 study will test two new dose levels of melphalan plus lenalidomide. The aims are to determine doses that can be safely administered without routine use of g-csf and to obtain estimates of efficacy that would justify proceeding to a randomized trial comparing this combination with the combination of melphalan, prednisone, and thalidomide. The MY.11 trial will be a randomized multi-centre, open-label, dose-finding, phase ii study testing two dose levels of lenalidomide plus melphalan.

In one arm of the trial, lenalidomide 15 mg daily will be given on days 1–21 of each 28-day cycle in combination with melphalan 4 mg/m^2^ given on days 1–4 of each cycle. The purpose of this arm is to test what ncic ctg believes to be the highest dose of lenalidomide that can be used as part of combination therapy. The dose of melphalan has therefore been proportionately reduced.

In the second arm, lenalidomide 10 mg daily will be given on days 1–21 of each 28-day cycle in combination with melphalan 6 mg/m^2^ given on days 1–4 of each cycle. This dose level is lower than that successfully tested by Palumbo (melphalan 0.18 mg/kg and lenalidomide 10 mg), but at the dose level used by Palumbo’s group, g-csf support was frequently required when those doses were tested. If dose-limiting toxicities are seen with lenalidomide 10 mg daily and melphalan 6 mg/m^2^, the dose of melphalan will be reduced to 5 mg/m^2^ in subsequent patients evaluated. The purpose of this arm is to further test a lower dose of lenalidomide with a proportionately higher dose of melphalan. Dose reductions of lenalidomide will be permitted beginning with cycle 1, and dose adjustments of melphalan will be allowed beginning with cycle 2. We believe that testing of these doses will complement the Italian trail and will provide, with a higher level of confidence, the optimum doses for phase iii testing.

In ncic ctg MY.7, a trial of standard-dose melphalan and prednisone, 31% of patients received a reduced dose of melphalan during cycle 2 or 3 of the drug. The myelosuppression of the combination of melphalan and lenalidomide is expected to be cumulative. Because melphalan is the accepted standard agent in the treatment of myeloma, maintaining the dose intensity of melphalan within an acceptable range when it is part of a combination is desirable. Given the apparently potent efficacy of lenalidomide and the potential for a dose–response relationship with this agent, the need to reduce the dose of melphalan from its standard 9 mg/m^2^ to 6 mg/m^2^ (with lenalidomide 10 mg) or to 4 mg/m^2^ (with lenalidomide 15 mg) appears to be appropriate. The MY.11 study will therefore be powered so that, for each starting dose of lenalidomide, the combination will have no further interest if the rate of dose reduction of melphalan or discontinuation of lenalidomide in cycle 2 or 3 is greater than 50%. Dose reductions of lenalidomide for hematologic toxicity will be permitted and will not be considered as dose-limiting toxicities unless associated with dose reductions of melphalan. The analysis of each treatment arm will then be intent-to-treat, based on each of the two dose levels.

Recent reports from the Southwest Oncology Group indicate that use of prednisone at a dose of 50 mg on alternate days significantly affects disease-free and overall survival after standard-dose chemotherapy, with minimal toxicity^17^. Specifically, disease-free survival improved from 6 months to 13 months and overall survival improved from 28 months to 43 months in 126 randomized patients. Similarly, dexamethasone maintenance is also of benefit as shown in ncic ctg MY.7, which demonstrated a clear benefit in progression-free survival for patients receiving dexamethasone maintenance therapy after melphalan and prednisone^18^. After 12 cycles, patients on the MY.11 protocol who have completed melphalan and lenalidomide and who have not progressed will be maintained on dexamethasone until progression or death and will be followed for progression-free survival.

### 2.3 Immunomodulating Drugs and Venous Thromboembolism

Treatment with thalidomide has been associated with an increased risk of venous thromboembolism. This risk appears to be especially present when the drug is combined with chemotherapeutic drugs such as melphalan and prednisone.

In the randomized trial reported by Palumbo^3^, more venous thromboembolism was observed in patients randomized to melphalan, prednisone, and thalidomide than in those randomized to melphalan and prednisone alone (12% vs. 2%, *p* = 0.001). This observation resulted in a protocol amendment partway through the period of patient accrual to mandate the use of enoxaparin.

Similarly, data from randomized trials have noted an increased risk of venous thromboembolism in patients treated with lenalidomide, including a potential increase in this risk when lenalidomide is combined with corticosteroids. For instance, in a randomized trial comparing lenalidomide plus dexamethasone with dexamethasone alone, an increase in venous thrombotic events was observed in the lenalidomide arm (15% vs. 3.5%, *p* value not stated)^19^.

Prophylactic therapy for venous thrombosis in myeloma patients treated with thalidomide or lenalidomide is therefore a topical issue. No randomized data are available to guide options that might include no prophylaxis or use of aspirin, warfarin, or heparin (fractionated or unfractionated). In the absence of such data, practice variation is observed between investigators, with many trials now including prophylaxis with either aspirin or low molecular weight heparin. In the phase i trial by Palumbo and colleagues that tested the addition of lenalidomide to melphalan and prednisone, aspirin 100 mg was included, and only 1 episode of venous thromboembolism was observed in 50 patients^16^.

Because the use of low molecular weight heparin could pose increased risk of bleeding and raises important issues of compliance, cost, and feasibility, treatment with aspirin may be a preferable alternative in these patients.

The role of aspirin in preventing venous thromboembolism is controversial. The most recent guidelines from an international panel recommend against the use of aspirin as prophylaxis, in part because of the proven superior efficacy of anticoagulation therapy in the types of patients evaluated^20^. However, other data suggest that aspirin, as compared with placebo, may be effective in preventing venous thromboembolism^21^,^22^. The MY.11 trial will not have sufficient statistical power to formally address this question in a comparative manner. Given the concerns regarding the routine use of anticoagulants in the relevant population and also given the initial data from Palumbo *et al.,* ncic ctg has chosen to include treatment with aspirin 81 mg as part of the therapeutic regimen of the pilot study.

## 3. SUMMARY

After many years of study and multiple clinical trials showing no advantage of newer combinations over melphalan and prednisone, 2006 was a very eventful year. Two trials demonstrated improved outcomes using melphalan, prednisone, and thalidomide. However, thalidomide is associated with many disadvantages, and a great deal of interest in the thalidomide analogue lenalidomide has arisen. Lenalidomide has been demonstrated to be effective as a single agent and in combination with dexamethasone. The toxicity profile of lenalidomide may have advantages in comparison with that of thalidomide. The ncic ctg has developed the MY.11 trial to investigate the dosing profile, tolerability, and efficacy of the combination of lenalidomide and melphalan for newly diagnosed myeloma patients. The study is designed to examine both short- and longer-term tolerability, and it includes the use of maintenance dexamethasone. With information gained from the MY.11 trial, a tolerable dosing schedule for the combination of melphalan and lenalidomide will be developed. That schedule will be used, in part, to inform the dosing levels and schedule for a phase iii randomized trial comparing melphalan, prednisone, and thalidomide with melphalan, prednisone, and lenalidomide. The new trial is being developed by the Eastern Cooperative Oncology Group and will include participation by ncic ctg.
